# Detection of Oral Fluid Stains on Fabric via Solution Extraction Combined with Deep Ultraviolet Raman Spectroscopy

**DOI:** 10.1021/acs.analchem.4c04581

**Published:** 2025-01-31

**Authors:** Alexis Weber, Mohamed O. Amin, Vladimir Ermolenkov, Entesar Al-Hetlani, Igor K. Lednev

**Affiliations:** † Department of Chemistry, University at Albany, SUNY, 1400 Washington Avenue, Albany, New York 12222, United States; ‡ Department of Chemistry, Kuwait University, Faculty of Science, P.O. Box 5969, 13060, Safat, Kuwait

## Abstract

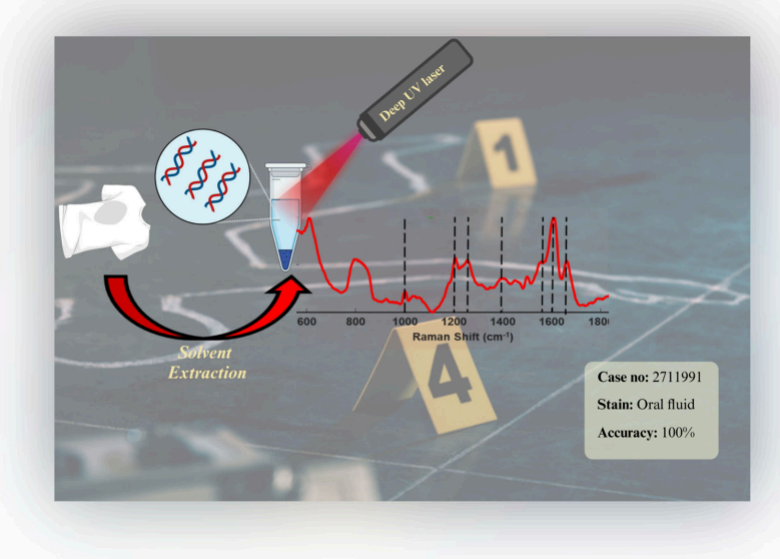

DNA phenotyping plays a central role in modern practical forensics, yet an overwhelming amount of evidence creates significant backlogs in all major crime laboratories. A fast nondestructive test of a potential biological stain prior to DNA phenotyping should reduce the number of irrelevant samples for the analysis and increase the efficiency of the overall process. Evidence items recovered from the crime scene can often include body fluid traces, such as oral fluid (OF). This proof-of-concept study demonstrates the effectiveness of Deep-UV Raman spectroscopy in identifying OF stains on substrates such as cotton, polyester, and blue denim, commonly encountered in forensic investigations. Through spectral interpretation and statistical analysis, this study compares Raman spectra from OF extracted from substrates with pure OF spectra. Additionally, comparative analysis with near-infrared (NIR) Raman spectroscopy to deep-UV Raman spectroscopy was performed. Distinct advantages of deep-UV Raman spectroscopy were determined, including reduced sample preparation requirements and the absence of fluorescence background, enhancement of the signal-to-noise ratio, and simplified data preprocessing. Using statistical analysis methods like principal component analysis and partial least-squares discriminate analysis, differentiation between OF and non-OF samples was possible. Overall, this study underscores the versatility and potential of deep-UV Raman spectroscopy as a valuable tool in forensic science.

## Introduction

The ability to detect and identify traces of body fluids can aid investigators in reconstructing the events of a crime scene and linking the assailant or victim to an incident. Verifying the type of traces from body fluids recovered from a crime scene is a ubiquitous process in many forensic investigations. This is due to the rich DNA content in body fluids. As with fingerprint (FP) comparison analysis, the results are considered unique to an individual and determined with a high degree of accuracy.^1,2^ However, before DNA analysis can be performed, ideally, the source of the body fluid trace should be determined.

A variety of presumptive and confirmatory tests are utilized by law enforcement agencies for detection and identification of body fluid traces.^3^ It is also critical to consider that body fluids are rarely found of satisfactory quality and quantity in the crime scene. This is because there are countless possible substrates on which the fluids might be deposited. These conditions will vary depending on the nature of crime, manner of transfer,^4^ the type of body fluid (DNA source),^4^ and the nature of the substrate (smooth, rough, porous, etc.).^5^ Therefore, it is of paramount importance to properly preserve the recovered traces of body fluids that will be used to perform presumptive and confirmatory tests without sacrificing the sample.

Evidentiary items recovered from the crime scene will often include traces of body fluids, particularly oral fluid (OF). OF evidence results from items such as bite marks, cigarette butts, stamps, envelopes, and other household items. The presence of OF can be difficult to detect, as dried OF stains are commonly present in trace amounts and are nonvisible in nature.^6,7^ The porosity of the substrate can also play a role in the detection process. Porous substrates tend to absorb OF, while in the case of nonporous substrates, the sample stays on the surface and can be exposed to variety of environmental conditions.^8^

The rapid and accurate identification of OF at crime scenes has become a field of interest for forensic case work to save time and resources and obtain affirmative answers. Locating OF evidence at the crime scene using nondestructive alternative light sources (ALS) equipped with different wavelengths to visualize fluorescence is a common practice. Though this technique has drawbacks due to its simplicity and the fact that it is not specific to OF.^9^ Furthermore, there is a possibility of fluorescence interference caused by the substrate which the sample deposited on.^8^ Laboratory-based tests for identifying body fluids include sensitive methods based on immunochromatographic- and enzyme-linked immunosorbent assays which detect the presence of α-amylase, an enzyme present in high concentrations in OF. However, these tests are destructive, influenced by environmental factors, and susceptible to false positive and negative results.^10^ Furthermore, detection of α-amylase utilizing the Phadebas test is not conclusive to saliva because the α-amylase enzyme is present in other body fluids in small amounts.^5,11^ Thus, while this is one of the primary techniques used for OF analysis, it is still highly presumptive in its conclusions.

Immunoassays also consume the sample, which can be problematic when evidence amount is limited. In contrast, deep-UV Raman spectroscopy addresses these challenges by providing highly specific molecular fingerprints without consuming the sample. This nondestructive approach allows for rapid, confirmatory identification, which reduces the reliance on immediately preforming DNA analysis when initial screening is not possible. Additionally, deep-UV Raman spectroscopy also avoids background fluorescence issues, making it an ideal tool for testing extractions from complex matrices like fabric substrates commonly encountered in forensic cases. By enabling a clear differentiation of biological stains from substrate materials, deep-UV Raman spectroscopy enhances sensitivity and practicality in ways that do not consume the sample and allow the saliva to be used for further analysis.

Owing to the destructive and inconclusive nature of these tests, forensic practitioners sometimes rely on DNA analysis of the body fluid traces, regardless of the stain origin, for identification purposes. The analysis process includes DNA extraction followed by quantification, amplification, and electrophoresis. For this process several reagents, apparatuses, and steps are involved which requires significant time and funding.^12^ This can sometimes result in unnecessary DNA testing for artificial substances, nonhuman samples, or analysis of samples that are not relevant to the crime. Erroneous examinations can cause large DNA tests backlogs and samples awaiting analysis risking degradation.^13−16^

In this preliminary study, we propose an alternative approach using deep-UV Raman spectroscopy and near-infrared (NIR) Raman spectroscopy following a simple extraction protocol for the identification of OF on different substrates. Raman spectroscopy (RS) has proven to be a powerful and high accuracy confirmatory tool in body fluid detection and identification.^17−22^ RS which relies on measuring inelastically scattered light of body fluids has demonstrated to be a rapid and nondestructive approach for analysis. RS signal can be obtained from a small sample size (a few fL is sufficient) and requires limited sample preparation. Moreover, deep-UV Raman spectroscopy is a special variant of RS which relies on the selective excitation of the sample and has received limited attention in the field of body fluid analysis for forensic applications.

In deep-UV Raman spectroscopy, the excitation wavelength falls within the analyte’s absorption band, and as a result, the intensity of the scattered beam is increased by a factor of up to 10^6^ times.^23,24^ The increased strength of the signal enables the detection of diluted samples (10^–7^ M), while selectively exciting a chromophore from deep within a complex matrix.^25,26^ Deep UV Raman specifically occurs when UV excitation is utilized at a wavelength below or around 200 nm to excite the specific chromophore in the sample while also minimizing the fluorescence band contribution.^27^ In the forensics domain, Deep-UV Raman spectroscopy has been reported for the detection of narcotics like heroin and cocaine and explosives including 4-mononitrotulene (4MNT), 3,4-dinitrotulene (34DNT), 2,4,6-trinitrotulene (TNT), pentaerythritol tetranitrate (PETN), and dimethyldinitrobutane (DMNB) using 244 nm excitation.^28^ Additionally, deep-UV Raman spectroscopy has been employed for the detection of cocaine in OF with the concentration down to 10 μg/mL without sample preparation and excitation using laser excitation of 239 nm.^29^

The utility of deep-UV Raman spectroscopy in identifying biological stains is noteworthy as it is a less common method of analysis. Compared to other techniques, such as traditional Raman spectroscopy, which often suffers from background fluorescence issues, deep-UV Raman spectroscopy effectively minimizes such interference. Thus, this allows for clearer spectra for the identification of biological materials. Though fluorescence spectroscopy on its own has previously been used for the identification of human saliva.^7^ Another common technique for biological fluid analysis is FTIR spectroscopy that has been shown to be viable for the identification of biological stains,^30^ however there is a risk of sample contamination when using the ATR attachment and requires more research before being useful for practical application. Overall, deep-UV Raman spectroscopy’s advantages in nondestructive analysis, high specificity, and limited sample preparation^27,29^ make it an ideal choice for screening biological stains in forensic investigations.

In this work, a comparative study using NIR Raman spectroscopy and deep-UV Raman spectroscopy for the analysis of OF stains was performed. Initially, OF components were extracted from three common substrates, including denim, cotton, and polyester, to emulate evidence found at crime scenes. Subsequently, the extracts were subjected to both NIR Raman spectroscopy and deep-UV Raman spectroscopy as a rapid and nondestructive approach to identify OF. The application of principal component analysis (PCA) on the data sets confirmed that there are differences between the OF controls and that of extracted OF samples. Furthermore, the external validation of the binary class partial least-squares discriminant analysis (PLSDA) model enabled a confirmatory discrimination between the OF extract samples and non-OF samples, obtained by conducting extraction from OF-free substrates. The developed approach could be potentially expanded to be more apt for forensic laboratories, in which an extract solution is initially tested prior to DNA analysis, to confirm the identity of the body fluid.

## Methods and Materials

### Sample Preparation

Three fresh oral fluid samples were collected from each of five donors, totaling 15 tubes, each containing 5 mL of oral fluid. Samples were collected by following a protocol approved by the Institutional Review Board (IRB) at the University at Albany. Before oral fluid collection, all donors signed written consent. This consent included an acknowledgment that donors were healthy adults and did not utilize prescription or recreational drugs. Donors were asked to refrain from eating or drinking for 1 h prior to the collection of the oral fluid. Substrates tested for this study included white cotton, white polyester, and blue denim. These are fabrics that are commonly encountered in criminal investigations in the cases of physical or sexual assaults. For samples that were to be exposed to oral fluid, three squares of each substrate were cut per donor and placed in individual Petri dishes. This meant 15 squares of cotton, polyester, and blue denim were prepared, totaling 45 samples.

For sample preparation, 200 μL of OF were deposited onto each substrate and allowed to dry for 24 h. Prior to deposition, oral fluid samples were vortexed to ensure homogeneity of the sample. Additionally, three blank samples were prepared for each substrate. These were fabric squares that did not have oral fluid deposited on the substrate but still underwent the extraction process. Oral fluid controls were prepared for each donor, consisting of a mixture in a 1:1 ratio of oral fluid and deionized (DI) water.

To extract the oral fluid from the substrates, the cuttings were first placed in an Eppendorf tube with 300 μL of DI water. Tubes were then shaken via a vortex for 10 min. The substrate cuttings were then removed from the tube and placed within a spin basket that was inserted into the same tube. The tube/spin basket combination was then centrifuged for 15 min to ensure that all liquid was removed from the fabric. The spin basket with the substrate was then stored back in the original Petri dish, while the resulting oral fluid solution was stored for analysis on a deep-UV Raman spectrometer. Samples were stored at 4 °C when not being tested on the Raman spectrometer.

For samples to be analyzed on the NIR Raman spectrometer, one additional sample preparation step was required. As the Raman spectrometer used does not have a liquid sample apparatus, 20 μL of the oral fluid extract or control samples were deposited onto an aluminum foil-covered slide and a cast film was created. The sample was then left to dry overnight under ambient conditions prior to sample collection.

### Sample Analysis

#### Deep-UV Raman Spectroscopy

A detailed description of a home-built deep-UV Raman spectroscopic apparatus can be found elsewhere.^31^ Briefly, a 197 nm fourth harmonic generation Indigo S laser system (Coherent, Inc.) was used for excitation. Raman scattering was dispersed and captured through a custom double monochromator connected to a liquid-nitrogen-cooled CCD camera (Roper Scientific, Inc.). A rotating Suprasil NMR tube equipped with a magnetic stirrer served as the sample holder. The obtained Raman spectra were preprocessed by using GRAMS/AI software from Thermo Electron Corp. to analyze spectral contributions, with water and quartz components being numerically subtracted prior to model building. For deep-UV Raman spectroscopy, the laser power on the sample was kept between 0.5 and 1.0 mW, while for NIR Raman, the laser power was 110–130 mW. A 197 nm laser beam (∼1 mW, Indigo-S laser system from Coherent) was focused onto a spinning Suprasil NMR tube (5 mm outer diameter, 0.38 mm wall thickness) containing 100-μL solution. To avoid photodegradation of the samples, the solutions were additionally mixed by using a magnetic stirrer. Scattered radiation was collected in backscattering geometry, dispersed using a homemade double monochromator, and detected with a liquid-nitrogen-cooled CCD camera (Roper Scientific). The accumulation time for each spectrum was 6 min.

#### NIR Raman Spectroscopy

Raman spectra were acquired using a Horiba XploRA Plus Confocal Raman Microscope, employing a 785 nm excitation laser set at 100% power and a 50× long working distance objective. Prior to each day’s experimental data collection, the instrument underwent calibration using a silicon standard (with a peak at 520.6 cm^–1^). Employing an automatic mapping stage, we collected 15 accumulations with 10 s exposure times at 5 map points, covering the spectral range of 300–1800 cm^–1^. These collection parameters were optimized to achieve a satisfactory signal-to-noise ratio, akin to what has been attained in prior studies. Data preprocessing and analysis were conducted using MATLAB (MathWorks, Inc.; version 9.3.0.713579, R2017b) equipped with PLS_Toolbox version 8.7 (eigenvector Research Inc.).

#### Chemometrics

For spectra collected on the deep-UV Raman spectrometer, preprocessing included truncating the spectra to 550–1850 cm^–1^, baseline correction using automatic weighted least-squares (AWLS) to the second order, normalization by total area, and smoothing using SavGol with a 15-filter width. Two models were built using these data. The unsupervised chemometric model, PCA, was used to determine how the spectra clustered and was created using three PCs. The binary PLSDA model was also created to distinguish between samples that possessed oral fluid from those that did not. This model was developed using four latent variables (LVs) and leave-one-out cross validation; samples from three donors were used to create the calibration data set, while the samples from the remaining two donors were used to build the validation data set.

The following preprocessing steps were applied to ensure accurate and consistent spectral data for chemometric modeling: baseline correction, normalization, and smoothing. Each step was chosen to address specific challenges inherent in spectroscopic data. Due to background signals and potential fluorescence, baseline correction was essential to isolate the relevant Raman bands from background interference. This step removed low-frequency variations, ensuring that the spectra represent only the molecular information from the sample.^32^ By applying an automatic weighted least-squares (AWLS) method, we achieved a clean baseline across samples, enhancing the accuracy of band identification, which is crucial for the subsequent chemometric models.

The next step was normalization by the total area. This was used to account for variations in the sample concentration and differences in the laser intensity across measurements. This process ensured that all spectra were on a consistent scale as normalization thus provided uniformity, making the spectral data comparable across samples.^32^ Finally, Savitzky–Golay smoothing was employed to reduce the high-frequency noise without distorting the spectral features. This step improved the signal-to-noise ratio, allowing the chemometric analysis to focus on true spectral patterns rather than random fluctuations.^32^ This was particularly important in deep-UV Raman spectra, where high sensitivity can amplify noise alongside the signal. Together, these preprocessing steps were chosen to maximize the chemometric model’s performance by enhancing spectral clarity and minimizing the influence of extraneous variables.

The preprocessing for the spectra collected using the NIR Raman spectra was based on a previous methodology for body fluid analysis. The previously created methodology by Muro et al. for the identification of five commonly encountered body fluids (peripheral blood, saliva, semen, sweat, and vaginal fluid) by Raman spectroscopy and chemometrics was employed for the identification of the oral fluid samples collected on the NIR Raman spectrometer.^33^ This methodology developed statistically confident models for the identification of body fluids. For this study, the Support Vector Machine Discriminant Analysis (SVMDA) model was selected, and the methodology for spectral analysis was followed. Preprocessing for the body fluid identification model, the spectra were truncated to 400–1700 cm^–1^ to remove any instrumental artifacts AWLS fifth order baseline corrected and normalized by total area and autoaligned per the instructions for the model. Additionally, a binary PLSDA model was created using these data using the same parameters as the deep-UV Raman data.

## Results and Discussion

### Analysis of Deep-UV Raman Spectra from Oral Fluid Extracts

**Figure 1:**
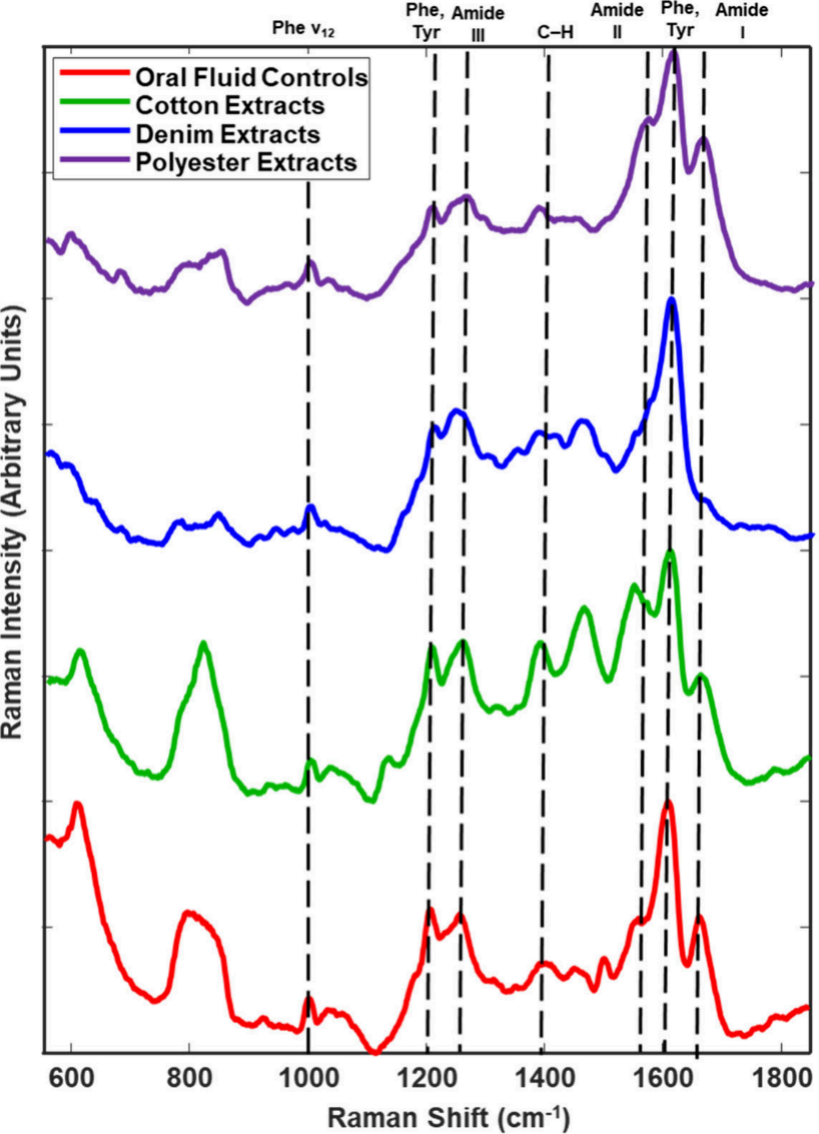
Stacked deep-UV Raman spectra of pure OF samples and OF extracted from cotton, polyester, and denim substrates. Bands of interest are marked with a dotted line, and band identification is denoted at the top of the figure.

Deep-UV Raman spectra were collected from liquid OF extracted from cotton, denim, and polyester. Visual examination of the spectra obtained from substrate OF extracts showed numerous similarities to those of the pure liquid oral fluid. The averaged spectra of the OF controls compared to the salivary extracts are shown in Figure 1. This indicates that the proposed method allows for the successful extraction of OF components on various substrates. Furthermore, clear distinctions were observed between the spectra of OF extracts and those of the substrate blank extracts across all substrates, affirming the specificity of the extraction process and the presence of identifiable OF markers.

Similarities between the OF controls and the OF extracts are denoted by the dashed lines on the corresponding Raman bands in Figure 1 and are present at 1000, 1200, 1255, 1388, 1464, 1609, and 1665 cm ^–1^. These bands have been previously assigned to various organic components in the literature.^27,31,34−36^ The band at 1000 cm ^–1^ is consistent with the vibrational mode of phenylalanine (Phe) v_12_, the band at 1400 cm ^–1^ was assigned to the vibration of C–H, and the bands at 1200 and 1609 are representative of Phe and tyrosine (Tyr). Finally, the bands at 1255, 1464, and 1665 cm ^–1^ were assigned to amide III, amide II, and amide I, respectively.^27,31,34−36^ While there were changes in the Amide I band in the spectra of the OF extracts from denim, this small change did not affect our ability to analyze and identify the sample as OF.

An exploratory statistical analysis of deep-UV Raman spectral data was done using the unsupervised approach PCA. A PCA score plot of the deep-UV Raman spectra of the OF controls, OF extracts from cotton, polyester, and denim, and extracts from the substrate blanks. These results are included in Supplementary Figure S1. Two PCA plots were prepared: one for the deep-UV Raman spectra and one for the NIR Raman spectra. Visual inspection of the deep-UV Raman spectra showed an overlap of the saliva controls with the saliva extracts from denim, cotton, and polyester. The blanks from the substrates were separated. From the PCA plot of the NIR Raman spectra, there were individual groupings of the saliva, with the saliva extracts from cotton, polyester, and denim also being separated. These differences in clustering between technique types are to be expected because deep-UV Raman spectroscopy and NIR Raman spectroscopy probe different chemical components in OF with these unique chemicals contributing to the overall spectrum in a different amount. Additionally, the analysis revealed no distinct separations within the OF extracts, indicating minimal variability between donors. This suggests that interdonor differences did not significantly impact the spectral characteristics or the classification outcomes in our study. The plot demonstrated distinct clustering of OF extracts with the OF controls, while substrate blanks showed no overlap with either the extracts or the OF controls. This separation highlighted the efficacy of deep-UV Raman spectroscopy in accurately distinguishing between the OF and non-OF samples. This suggested that there were significant differences between the deep-UV Raman spectra of OF and those of blanks extracted from the substrates.

To develop a statistical model for accurate discrimination, a supervised statistical approach based on the PLSDA was used. A binary PLSDA model was developed to distinguish deep UV Raman Spectra as either OF or non-OF. Remarkably, external validation of this model achieved a 100% accuracy rate, affirming its reliability in accurately identifying the classification of spectral data, shown in Table 1. Thus, showing that deep-UV Raman spectroscopy is paired with chemometric analysis, one is able to identify OF stains after being extracted from common substrates.

**Table 1:**
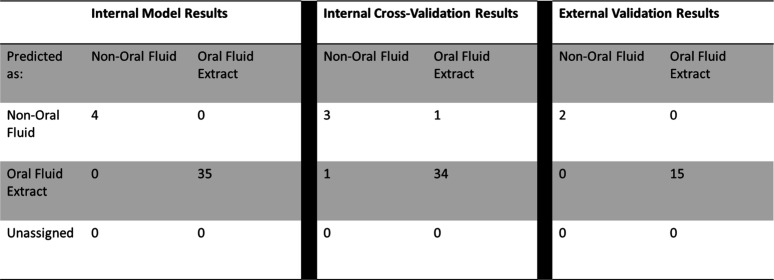
PLSDA Model Results for the Binary Model Discriminating between OF Extracts and Blank Samples from Deep-UV Raman Spectra^a^

a Tables include the internal model, internal cross-validation, and external validation results.

### NIR Raman Spectra Analysis of Samples from Oral Fluid Extracts

**Figure 2:**
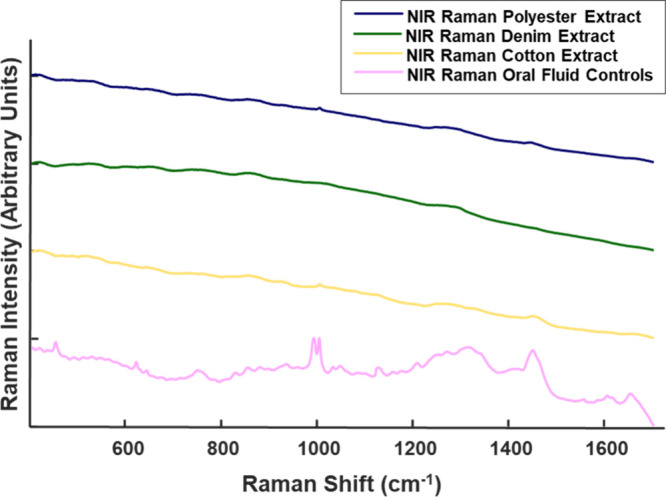
Raw NIR Raman spectra of OF extracts from substrates and pure oral fluid.

NIR Raman spectral analysis was also performed on the cast films from OF extracts from samples deposited on cotton, denim, and polyester. The resulting spectra had a large fluorescence background, as shown in Figure 2 and required preprocessing for the relevant Raman bands to be visually apparent. In comparison, while the OF control sample also had significant fluorescence background, the Raman bands for that sample were easily visible in raw spectra when compared to the extracted samples. Unlike NIR Raman, deep-UV Raman spectra have no fluorescence contribution since the latter occurs at longer wavelengths than those of the inelastic scattering. The excitation of the samples occurred below a wavelength that would cause fluorescent background interference.^37^

**Figure 3:**
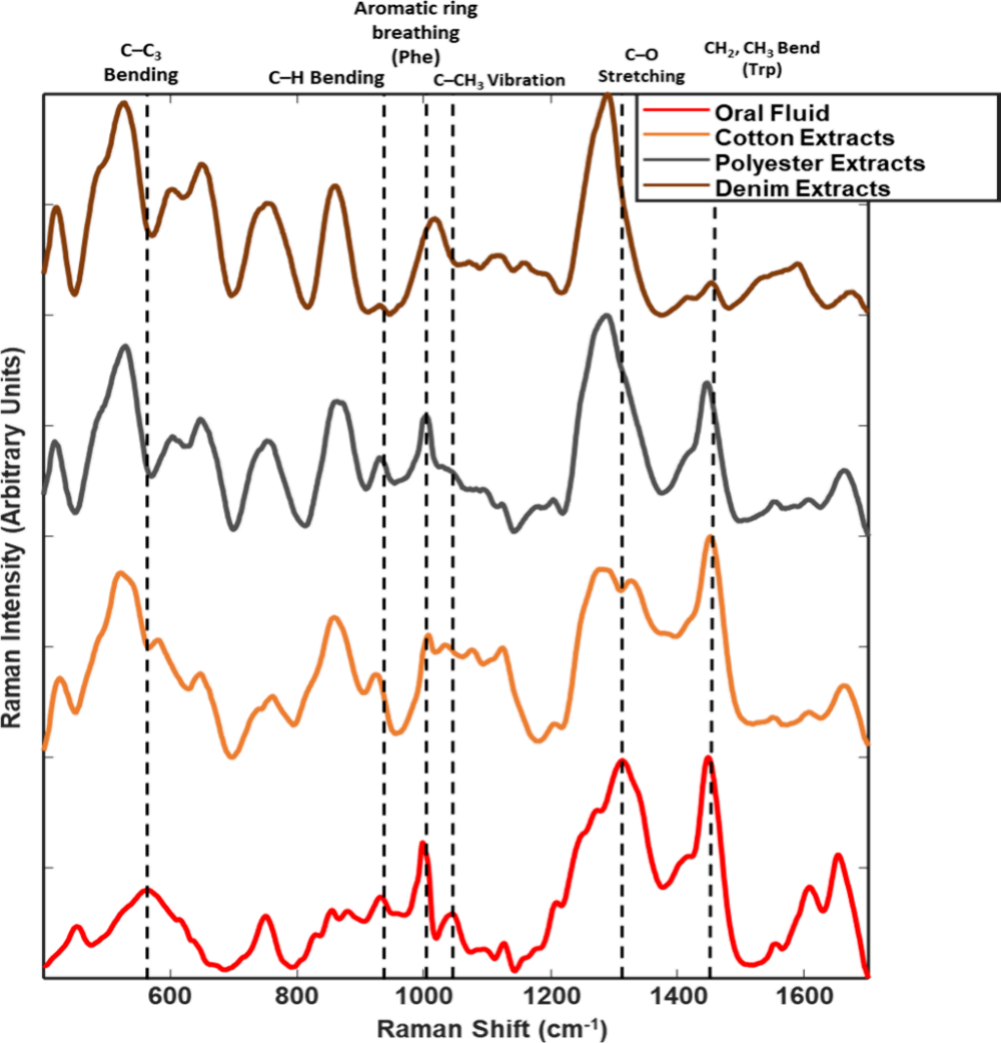
Stacked NIR Raman spectra of cast films from pure OF samples and OF extracted from cotton, polyester, and denim substrates. Bands of interest are marked with a dotted line and band identification is denoted at the top of the figure.

However, after preprocessing, the relevant Raman bands present within the spectra became more apparent. The average preprocessed spectra of the OF extracts are shown in Figure 3. This allowed for the comparison between the OF samples that were extracted from the three substrates and the control OF sample. When compared to the NIR Raman spectra of the OF control, the extracted samples possessed the same spectral bands. This includes the peak at 1449 cm^–1^ which was attributed to C–H_2_ and C–H bending in tryptophan (Trp).^19,38^ Other bands include those at 930, 1000, and 1070 cm^–1^ which based on previous work were assigned to C–H bending, aromatic ring breathing in Phe, and C–CH_3_ vibrations, respectively.^19,38^ Visually, the extracted samples were consistent with the sample of pure OF, but to allow for accurate discrimination, the use of chemometrics is needed.

A PCA scores plot was created to show the clustering of the NIR Raman spectra for the OF extracts from cotton, denim, and polyester compared with pure saliva. The PCA plot for NIR Raman spectra established that there was clustering of the OF extracts, both between each class (OF vs non-OF) of samples and between those of the OF controls. The resulting PCA scores plot is depicted in Supplementary Figure 1B. This is different from the PCA plot created from the deep-UV Raman spectra, where the OF controls were similar to those of the OF extracts. These results indicate that there was better separation observed with PCA when using NIR Raman spectra. To develop a classification model, a supervised PLSDA was created for discrimination of OF samples extracted from common substrates.

In previous work, the Lednev lab has shown the usability of PLDSA, SVMDA, and Random Forest models for the identification of various body fluids including blood, semen, saliva, sweat, urine, and vaginal fluid.^33,39−41^ The ultimate goal is to create a universal classification model developed for all main body fluids based on NIR Raman spectroscopy that is capable of working in all conditions. For instance, blood samples exposed to variable conditions such as increased time since deposition and heated aging have been tested within this model and have shown high accuracy for confirmatory identification of the spectra.^42−44^ Therefore, one of the goals of this work was to determine if this model was usable for the identification of the OF extracts from cotton, denim, and polyester. When tested on the SVMDA identification model, the spectra of the extracted OF stains were primarily grouped into the “unassigned” category, as shown in Table S1. These results have shined a light on a limitation of this previously developed model, that it requires additional development to work with samples that have been extracted from a substrate. This was not surprising as all samples tested in that model previously were not extracted from substrates but were either pure samples or spectra collected directly on the substrate.^8,45−48^

As a next step, a binary PLSDA model was created by comparing the NIR Raman spectra of the deposited OF extracts and blank samples (non-OF). Similar to the binary PLSDA model created for the deep-UV Raman spectra, external validation of this model achieved a 100% accuracy rate, shown in Table 2. Therefore, while the existing identification model was unable to properly identify the spectra from the saliva extracts, the use of PLSDA allows for accurate discrimination between the OF extract sample and non-OF samples.

**Table 2:**
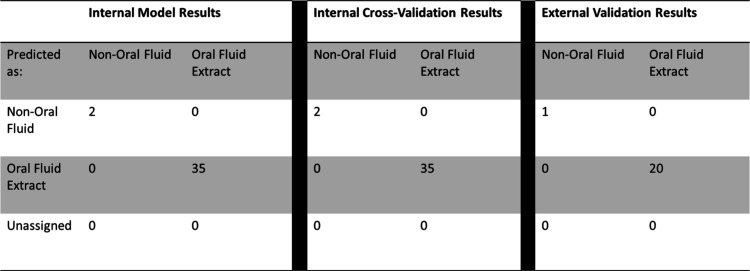
PLSDA for the Binary Model of OF Extracts Compared with Blank Samples Collected on the NIR Raman Spectrometer

### Comparison Between Deep-UV and NIR Raman Spectra

For this study, two spectroscopic methods were tested for the analysis of OF samples extracted from cotton, denim, and polyester substrates. In our study, the binary PLSDA model achieved 100% accuracy in distinguishing between OF extracts and non-OF samples for both deep-UV and NIR Raman spectroscopic techniques. This classification accuracy was based on a total of 45 samples from five donors. External validation was conducted using leave-one-out cross-validation to ensure the model’s robustness and to reduce potential overfitting.

To further support the model’s performance, we calculated additional metrics, including sensitivity, specificity, and statistical power. Sensitivity and specificity were both at 100%, indicating the model’s strong ability to correctly classify OF stains while avoiding false positives. The high statistical power demonstrates the model’s reliability across samples. These findings validate the robustness of the PLSDA models for both spectroscopic techniques but also highlight the practical advantages of deep-UV Raman for forensic applications, where high sensitivity and specificity are critical. In further research, the number of donors will be expanded to further test the effects of intra- and interdonor variability on the results.

Deep-UV Raman spectra exhibited satisfactory signal-to-noise ratios without the need for extensive sample preparation, thereby saving valuable time in the analysis process. Moreover, the raw deep-UV Raman spectra required minimal preprocessing for the identification of significant peaks and subsequent chemometric analysis, further streamlining the analytical workflow. The absence of fluorescence interference when analyzed on the deep-UV Raman spectrometer, unlike the NIR Raman spectrometer, contributed to higher quality spectra with enhanced interpretability and accuracy. We observed that deep-UV Raman spectroscopy excitation, while powerful, did not cause noticeable degradation to the sample or the spectra within the exposure limits used in this study when consecutive accumulations were acquired. Similarly, saliva analyzed with NIR excitation showed no form of degradation, although the fluorescence background in NIR could obscure fine spectral details, unlike deep-UV Raman spectroscopy.

Conversely, solutions of OF extracts cannot be directly analyzed using NIR Raman spectroscopy because of a low concentration of OF components, necessitating the preparation of cast films to enhance the signal-to-noise ratio of the spectra. This difference emphasizes the practical advantages of deep-UV Raman spectroscopy in forensic applications, as less sample preparation is required. Overall, the front end of analysis for deep-UV Raman spectroscopy is more suitable for integration into the forensic workflow, as it required less sample preparation and produced higher quality raw spectra. Integrating deep-UV Raman spectroscopy into forensic workflows can assist in streamlining evidence processing, adding an efficient, nondestructive step before DNA testing. By using deep-UV Raman spectroscopy as a preliminary screening method, forensic laboratories pretest samples with an unknown probability of containing relevant biological materials, allowing DNA testing resources to be allocated more strategically. In current practices, all potential samples often undergo DNA analysis, even when biological relevance is uncertain, which contributes significantly to the case backlogs. Incorporating deep-UV Raman spectroscopy as a rapid screening technique can alleviate these bottlenecks by allowing forensic scientists to confirm or exclude samples as relevant more quickly and accurately.

For the identification of the OF extracts using NIR Raman spectroscopy, challenges arose when attempting to identify OF samples extracted from substrates using the existing body fluid identification model. The extracted OF samples were classified as unassigned instead of as the proper body fluid. Consequently, the development of an additional binary model was necessary to differentiate between the OF and blank samples. The deep-UV Raman spectra were unable to be tested in this model and, thus, are not completely comparable. However, this highlights the importance of adapting analytical approaches to accommodate the complexities of real-world forensic scenarios.

In summary, this study investigated two spectroscopic methods for analyzing OF samples extracted from cotton, denim, and polyester substrates. Deep-UV Raman spectroscopy proved more advantageous for spectral collection and analysis compared with NIR Raman spectroscopy. While challenges were encountered in the identification of OF extracts using a universal classification model developed for all main body fluids based on NIR Raman spectroscopy, the development of additional analytical models highlighted the adaptability required for forensic applications. Visible excitation, while commonly used in Raman spectroscopy, typically encounters a fluorescence background from substrates or sample matrices, which can reduce spectral clarity. However, visible excitation could offer benefits in terms of lower cost and ease of implementation compared to those of deep-UV Raman systems. Future studies could explore visible excitation as a complementary approach. Moving forward, the integration of deep-UV Raman spectroscopy into forensic workflows offers a promising avenue for enhancing the efficiency and accuracy of biological evidence analysis, especially because deep-UV Raman spectroscopic instruments are commercially available.

## Conclusions

This proof-of-concept study presents compelling evidence for the effectiveness of deep-UV Raman spectroscopy in the precise identification of OF stains deposited on various everyday substrates, such as cotton, polyester, and blue denim. Samples collected at crime scenes may consist of materials such as t-shirts, jeans, or underwear. It is imperative that forensic analysts have multiple methods for analyzing samples on varying substrates to obtain the most probative information. The findings in this work highlight the potential of deep-UV Raman spectroscopy as a valuable tool in forensic science for the detection and analysis of biological evidence, offering promising avenues for enhancing forensic investigations and aiding law enforcement efforts.

Through spectral interpretation and statistical analysis, it was determined that the Raman spectra of extracted samples exhibit consistency with the characteristic spectrum of OF when scrutinized by using deep-UV Raman spectroscopy. The results depicted in this work emphasize this consistency, showcasing that the Raman spectra of the OF extracts align closely with those of the OF controls for the given samples. Utilizing advanced statistical techniques such as PCA and PLSDA, the study achieves a remarkable milestone by confidently identifying the deep-UV Raman spectra of OF extracts postdeposition on common substrates with an accuracy rate of 100%. This enhances the reliability of deep-UV Raman spectroscopy as a forensic tool, offering forensic scientists and law enforcement agencies a robust method for discerning biological evidence, even in complex scenarios.

Moreover, when compared to NIR Raman spectroscopy, deep-UV Raman spectroscopy emerged as a better choice for spectral analysis. While both techniques demonstrated their effectiveness for the analysis of OF stains, deep-UV Raman spectroscopy exhibits distinct advantages over its NIR counterpart. These advantages include fewer sample preparation requirements prior to analysis, as a cast film was required for testing only when using the NIR Raman spectrometer. Additionally, when using the deep-UV Raman spectrometer, there is no fluorescence background, unlike NIR Raman, resulting in spectra with a higher signal-to-noise ratio and requiring less preprocessing required prior to spectral analysis. Based on these results, deep-UV Raman spectroscopy shows valuable attributes for forensic applications, where precise identification and analysis are paramount.

However, it is essential to acknowledge the limitations of deep-UV Raman spectroscopy to provide a balanced view of its applicability. This study employed a home-built spectrometer, which while effective in this research setting, raises concerns regarding reproducibility and scalability for laboratories without access to custom-built equipment. Although commercial deep-UV Raman instruments are emerging, more work is needed to standardize this technology to ensure consistent results across various laboratory setups. Future research should explore the feasibility of using commercially available deep-UV Raman systems, such as the Photon Systems deep-UV Raman instrument (https://photonsystems.com/products/lab-spectrometer-systems/raman-pl200/), and examine reproducibility across different instruments and operators. Addressing these limitations would improve the method’s scalability and make it accessible to a broader forensic community.

Further research should also focus on expanding the forensic applications of deep-UV Raman spectroscopy by testing other biological fluids, such as blood and semen, across diverse substrates and exploring its sensitivity limits in detecting highly diluted stains. Additionally, developing commercially available desktop deep-UV Raman setups for more reproducible analysis in crime laboratories could offer a practical solution that reduces the need for sample transportation to specialized laboratories and preserves evidence integrity.

That is not to say that NIR Raman spectroscopy is not invaluable in its ability to analyze OF sample extracts. Using a PLSDA binary model, the samples were able to be distinguished from non-OF samples. This is similar to the results produced by the PLSDA binary model of deep-UV Raman spectroscopy. However, when the OF extracts were tested in our existing SVMDA body fluid identification model, most of the spectra were classified as unassigned. This indicates that more work is needed for the identification of stains extracted from the substrates. Future studies to expand on this proof-of-concept study include increasing the number of donors and substrates examined, testing this method for additional body fluid traces, and determining the limit of detection of OF extracts that can be identified using both deep-UV and NIR Raman spectroscopy.

Overall, this study highlights the versatility of deep-UV Raman spectroscopy by successfully detecting OF traces on cotton, polyester, and blue denim, which are commonly encountered during forensic investigations. Additionally, this comparative analysis further reinforces the potential of deep-UV Raman spectroscopy as an invaluable asset in forensic investigations. While there are scalability challenges to address, deep-UV Raman spectroscopy presents a promising tool that could enhance the efficiency and accuracy of forensic workflows. With continued research to standardize and validate its use across different laboratories, deep-UV Raman spectroscopy has the potential to become an invaluable resource for modern forensic science.

## Supporting Information Available

PCA Scores plot for all spectra collected and confusion matrix of NIR Raman spectra on a previous identification model (PDF)
