# Supplementary material for: Detection of Oral Fluid Stains on Fabric via Solution Extraction Combined with Deep Ultraviolet Raman Spectroscopy
Source: Anal Chem. 2025 Jan 31;97(7):3864–71. doi: 10.1021/acs.analchem.4c04581 (PMC11866284; doi:10.1021/acs.analchem.4c04581)
Supplement: Supplementary file 1 — ac4c04581_si_001.pdf [file ac4c04581_si_001.pdf]

Supplementary Information

Detection of oral fluid stains on fabric via solution extraction combined with deep ultraviolet Raman spectroscopy.

Authors: Alexis Weber[1], Mohamed O. Amin[1], Vladimir Ermolenkov[1], Entesar Al-Hetlani[2], and Igor K. Lednev[1*]

Affiliations:

[1]Department of Chemistry, University at Albany, SUNY, 1400 Washington Avenue, Albany, NY, 12222, USA

[2]Department of Chemistry, Kuwait University, Faculty of Science, P.O. Box 5969, 13060, Safat, Kuwait

Table of Contents

Figure S1 – PCA Scores plot of spectra collected from the deep-UV Raman spectrometer (A) and the NIR Raman spectrometer (B).

Table S1 – Spectra of extracts collected on NIR Raman spectra tested on a previously developed body fluid identification model.

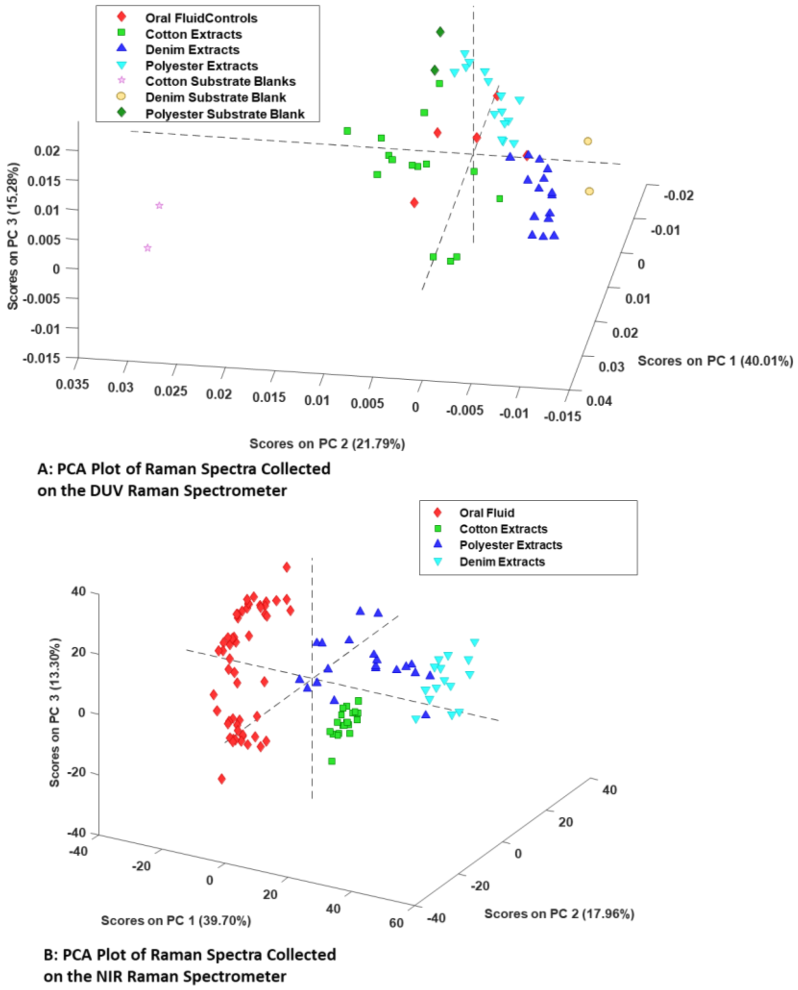

Figure S1: PCA Scores plot of spectra collected from the deep-UV Raman spectrometer (A) and the NIR Raman spectrometer (B).

Table S1: Spectra of extracts collected on NIR Raman spectra tested on a previously developed body fluid identification model.

**Predicted as**	**Saliva**
Peripheral Blood	0
Saliva	1
Semen	0
Sweat	1
Vaginal Fluid	0
Unassigned	53
Prediction Accuracy	2%
